# Roflumilast reverses CFTR-mediated ion transport dysfunction in cigarette smoke-exposed mice

**DOI:** 10.1186/s12931-017-0656-0

**Published:** 2017-09-18

**Authors:** S. Vamsee Raju, Lawrence Rasmussen, Peter A. Sloane, Li Ping Tang, Emily Falk Libby, Steven M. Rowe

**Affiliations:** 1 Department of Medicine, University of Alabama at Birmingham, Birmingham, AL USA; 2 Department of Pediatrics, University of Alabama at Birmingham, Birmingham, AL USA; 3 Department of Cell, Integrative, and Developmental Biology, University of Alabama at Birmingham, Birmingham, AL USA; 4 UAB Lung Health Center, University of Alabama at Birmingham, Birmingham, AL USA; 5 Cystic Fibrosis Research Center, University of Alabama at Birmingham, Birmingham, AL USA; 6 MCLM 702, 1918 University Blvd, Birmingham, AL 35294-0006 USA

**Keywords:** CFTR, Roflumilast, cAMP, COPD, Chronic bronchitis

## Abstract

**Background:**

Dysfunction in cystic fibrosis transmembrane conductance regulator (CFTR) can be elicited by cigarette smoke and is observed in patients with chronic bronchitis. We have previously demonstrated in human airway epithelial cell monolayers that roflumilast, a clinically approved phosphodiesterase 4 inhibitor that reduces the risk of exacerbations in chronic obstructive pulmonary disease patients with chronic bronchitis and a history of exacerbations, activates CFTR-dependent chloride secretion via a cAMP-mediated pathway, partially restores the detrimental effects of cigarette smoke on CFTR-mediated ion transport, and increases CFTR-dependent gastrointestinal fluid secretion in isolated murine intestine segments. Based on these findings, we hypothesized that roflumilast could improve CFTR-mediated chloride transport and induce secretory diarrhea in mice exhibiting cigarette smoke-induced CFTR dysfunction.

**Methods:**

A/J mice expressing wild type CFTR (+/+) were exposed to cigarette smoke or air with or without roflumilast and the effect of treatment on CFTR-dependent chloride transport was quantified using nasal potential difference (NPD) measurements in vivo and short-circuit current (Isc) analysis of trachea ex vivo. Stool specimen were collected and the wet/dry ratio measured to assess the effect of roflumilast on secretory diarrhea.

**Results:**

Acute roflumilast treatment increased CFTR-dependent chloride transport in both smoke- and air-exposed mice (smoke, −2.0 ± 0.4 mV, 131.3 ± 29.3 μA/cm^2^, *P* < 0.01 and air, 3.9 ± 0.8 mV, 147.7 ± 38.0 μA/cm^2^, *P* < 0.01 vs. vehicle −0.3 ± 0.7 mV, 10.4 ± 7.0 μA/cm^2^). Oral administration of roflumilast over five weeks completely reversed the deleterious effects of cigarette smoke on CFTR function in smoke-exposed animals, in which CFTR-dependent chloride transport was 64% that of air controls (roflumilast, −15.22 ± 2.7 mV vs. air, −14.45 ± 1.4 mV, *P* < 0.05). Smoke exposure increased the wet/dry ratio of stool specimen to a level beyond which roflumilast had little additional effect.

**Conclusions:**

Roflumilast effectively rescues CFTR-mediated chloride transport in vivo, further implicating CFTR activation as a mechanism through which roflumilast benefits patients with bronchitis.

## Background

Cystic fibrosis transmembrane conductance regulator (CFTR) is a cyclic adenosine monophosphate (cAMP)-regulated chloride channel apically expressed on epithelial cells in the lung, nose, and intestine, among other tissues [1]. Genetic defects in CFTR are well known for their causative role in cystic fibrosis (CF), and are linked to the pathogenesis of an array of other disorders including idiopathic bronchiectasis [2, 3], chronic sinusitis [4], allergic bronchopulmonary aspergillosis [5], congenital bilateral absence of the vas deferens [6], and recurrent idiopathic pancreatitis [7, 8]. It is increasingly appreciated that acquired CFTR dysfunction in the absence of congenital mutations also contributes to the pathophysiology of human diseases. Prominently, acquired CFTR dysfunction has been independently linked to chronic obstructive pulmonary disease (COPD) [9–12], the third leading cause of death in the US and source of over $30 billion in annual healthcare costs [13]. This association has been observed for the chronic bronchitis, versus emphysematic, form of COPD that exhibits features including mucin hyperexpression, mucus accumulation, and goblet cell hyperplasia [14] that induce impaired mucociliary clearance, chronic bacterial colonization and persistent neutrophilic airway inflammation [15], pathophysiology resemblant to CF lung disease.

We and others have demonstrated that acquired CFTR dysfunction can be instigated by cigarette smoke, the predominant risk factor for development of COPD. In airway epithelial cells, cigarette smoke has been shown to reduce CFTR mRNA and protein levels [16]; elicit CFTR internalization [17]; inhibit CFTR-dependent chloride transport [18]; and act acutely to decrease CFTR open-channel probability and chronically to partially reduce cell surface CFTR expression. These effects are reflected in clinical observations showing that CFTR-mediated ion transport is compromised in the nasal [10, 16] and lower airways [12], sweat gland [9, 11], and intestine [9] of cigarette smokers both with and without COPD. As such, restoration of impaired CFTR function may represent an important approach in COPD therapeutics, as underscored by investigations exhibiting that the CFTR potentiator ivacaftor rescues CFTR ion transport and epithelial function in smoke-exposed human airway cells [10] as well as improves CFTR activity in current and former smokers with chronic bronchitis [19].

In further support of the significance of CFTR activation as a treatment modality for COPD, we have previously reported the CFTR-activating properties of roflumilast [20], a currently approved type 4 cyclic nucleotide phosphodiesterase (PDE4) inhibitor that reduces exacerbations in patients with chronic bronchitis and a history of frequent exacerbations [21]. The mechanistic basis behind the clinical benefits of roflumilast in COPD patients, including its relative predilection for individuals with frequent exacerbations and chronic bronchitis, characteristics potentially attributable to CFTR abnormality, have remained largely undefined. We found in primary human bronchial epithelial monolayers that roflumilast activated CFTR-dependent chloride secretion via cAMP/protein kinase A (PKA)-dependent phosphorylation of the CFTR regulatory domain, and was able to partially restore the detrimental effects of cigarette smoke on CFTR-mediated ion transport. Roflumilast also elicited CFTR-dependent gastrointestinal fluid secretion in murine intestine segments, suggesting a mechanism underlying the diarrheal side effects associated with roflumilast treatment [22].

In these studies, we evaluated the effect of roflumilast on CFTR activity in vivo to afford additional evidence demonstrating the relevancy of the CFTR pathway in mediating the effect of roflumilast, specifically, and as a treatment approach for COPD, more broadly. We established that roflumilast stimulates CFTR-dependent ion transport in healthy and smoke-exposed mice, and ameliorates smoke-inducted CFTR dysfunction. We also observed that roflumilast triggers secretory diarrhea in a smoke-independent manner, potentially informing a strategy to minimize this adverse event.

## Methods

### Animal studies

All animal protocols were reviewed and approved by the University of Alabama at Birmingham Institutional Animal Care and Use Committee. Gender-matched 6–9 week-old A/J mice expressing wild type CFTR (+/+) were used for all studies. Mice were exposed in whole-body chambers (28″ × 19″ × 15″) to diluted mainstream cigarette smoke (up to 200 μg/l of total particulate matter, 35-ml puffs of 2-s duration at a rate of 3 L/s each minute for 40 min) from 3R4F reference cigarettes (University of Kentucky, Lexington, KY) twice daily for five weeks using an automated cigarette smoking apparatus (SCIREQ, *InExpose* model, Toronto, Canada). Control mice were exposed to room air in same-sized chambers. Characterizations of whole cigarette smoke exposures (e.g., volumetric flow rate calibration, aerosol concentration, particle size distribution) were previously reported [9].

### Treatment protocol

Roflumilast stock solution was prepared in PEG400 by continuous stirring in a 70 °C water bath. Once dissolved, the stock solution was diluted with equal proportions of 4% methocel E15 (Dow Chemicals, Delaware) and stirred continuously until a homogenous milky suspension was obtained. Where indicated, mice were administered roflumilast (5 mg/kg/d) or vehicle for five weeks by oral gavage (maximum volume of 10 ml/kg/d).

### Measurement of CFTR activity

CFTR function was assessed in murine nasal epithelium by nasal potential difference (NPD) measurements and in murine trachea by analysis of short-circuit current (Isc) according to previously published methods [9]. Briefly, for NPD assessment, mice were anesthetized and sequentially perfused with Ringer’s solution (baseline); Ringer’s plus amiloride (100 μm) and a chloride-free solution containing K_2_HPO_4_ (2.4 mM), KH_2_PO_4_ (0.4 mM), Na Gluconate (115 mM), NaHCO_3_ (25 mM), and Ca_2_ Gluconate (1.24 mM) with forskolin (20 μM); and roflumilast (30 nM). CFTR-dependent chloride transport was measured as the change in potential difference following perfusion with chloride-free ringers followed by forskolin or by CFTR-specific inhibitor cocktail containing 10 μM each of GlyH101 and CFTR_inh_-172.

Mice were euthanized and trachea were harvested and tested as full-thickness tissue. Isc was measured under voltage clamp conditions using P2300 Ussing chambers and MC8 Voltage Clamps (Physiologic Instruments, San Diego, CA). Mounted tissues were bathed on both sides with identical Ringers solutions gassed with 95% O_2_:5% CO_2_ and then sequentially treated apically with amiloride (100 μM), roflumilast (30 nM), ATP (100 μM), and bumetanide (10 μM; added only to the serosal solution at the end of experiments to block chloride ion-dependent Isc).

### Assessment of diarrhea

Drug-induced diarrhea was monitored qualitatively by stool output and presence of perianal staining of the coat. Severity of diarrhea was estimated in fresh stool specimen collected on a clean surface outside the cages by determining the water content via measurement of the wet-to-dry ratio. Dry weight was calculated after samples were air-dried at 65 °C for 24 h.

### Statistics

Data were analyzed using Student’s t-test or ANOVA, with post-hoc tests as appropriate. All statistical tests were two-sided with a *P*-value of 0.05 demarcating significance, and were conducted using GraphPad Prism software (La Jolla, CA). Data is reported as mean ± SEM, unless otherwise notated.

## Results

### Roflumilast increases CFTR activity in the nasal airways in vivo and tracheal segments ex vivo

**Fig. 1:**
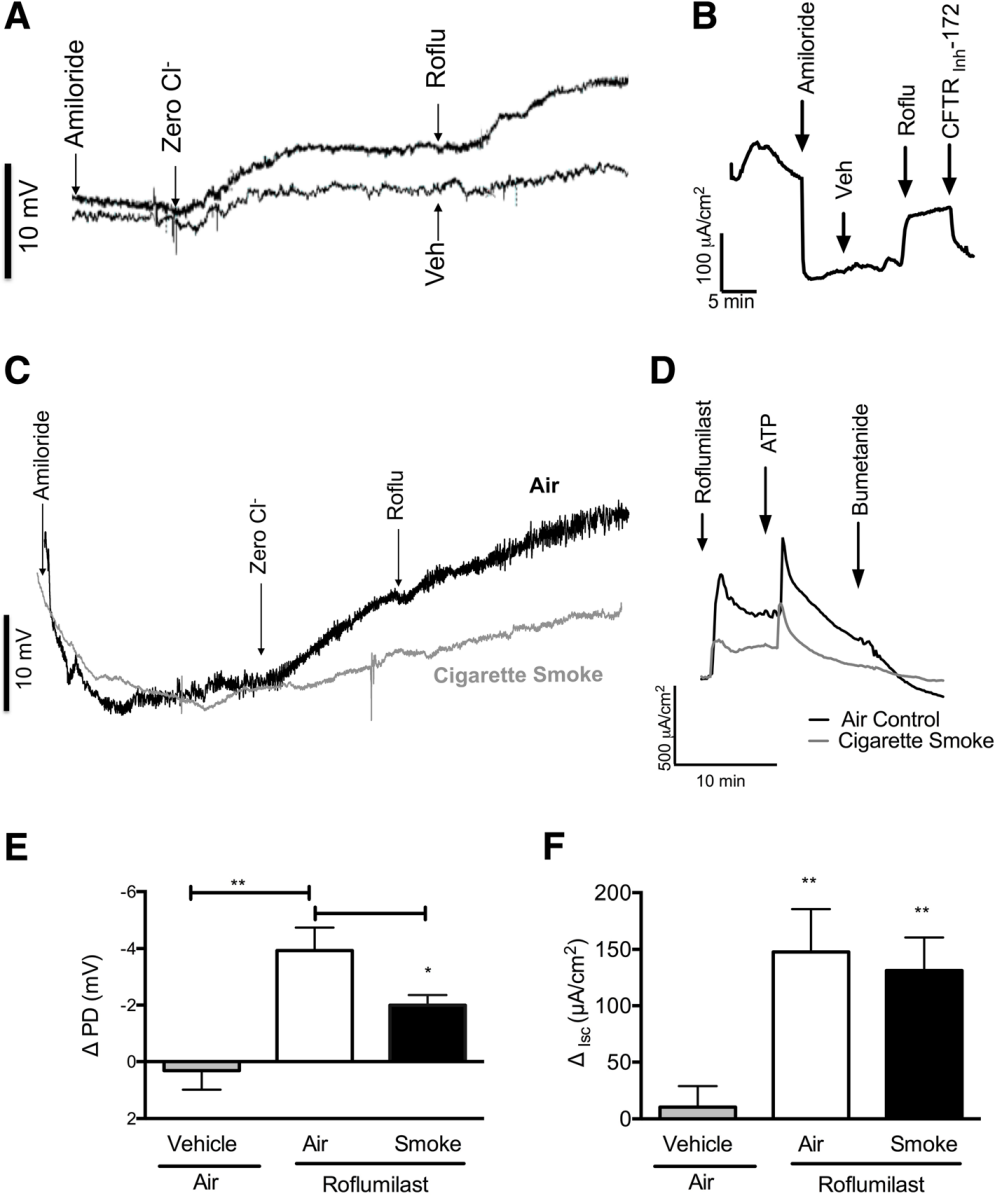
Effect of acute roflumilast perfusion in normal and cigarette smoke-exposed mice. **a**, **b** Non-CF A/J mice were treated with roflumilast (30 nM) or vehicle perfused acutely on the nasal surface *N* = 7–9. **a** Representative nasal potential difference (NPD) tracings of roflumilast as compared to vehicle following perfusion with amiloride (100 μM) and forskolin (20 μM). **b** Representative short-circuit current (Isc) measurements of excised mouse trachea treated with vehicle followed by roflumilast (30 nM) in the setting of amiloride (100 μM). CFTR-_Inh_172 (10 μM) was added to block CFTR-specific current at the end of the experiment. **c**-**f** Non-CF A/J mice were exposed to cigarette smoke or air via a nose-only exposure system for five weeks, and treated with roflumilast (30 nM) or vehicle acutely onto the airway surface, *N* = 5–7. **c**, **d** Representative NPD tracings (**c**) and tracheal Isc measurements (**d**). **e** Mean roflumilast-stimulated change in NPD following sequential perfusion of Ringer’s solution, Ringer’s solution with amiloride (100 μM), and chloride-free Ringer’s alone, *N* = 7–15, *P < 0.05, **P < 0.01. **f** Mean change in Isc upon treatment with roflumilast (30 nM) in the setting of amiloride (100 μM). ATP (100 μM) was added to induce chloride secretion through CFTR-independent channels; bumetanide (10 μM) was used to block chloride ion-dependent Isc. mice/condition. *P < 0.05, **P < 0.01. Roflu = roflumilast, Veh = vehicle

To verify prior studies in airway monolayers and tissues that implicated CFTR activation as a mechanism mediating the clinical benefit of roflumilast [20], we evaluated whether roflumilast alters CFTR function on the surface of the respiratory epithelium in healthy, wild-type mice, the anticipated CFTR genotype in the majority of COPD patients [23]. To accomplish this, roflumilast (30 nM) or vehicle was acutely infused onto the nasal surface of non-CF A/J mice and NPD measurements were performed to quantify changes in voltage across the nasal epithelium due to altered transepithelial chloride transport [24]. As expected based on our previous observation that roflumilast activated CFTR in normal human bronchial epithelial cells [20] and as illustrated by the representative NPD tracing shown in Fig. 1a, chloride transport increased upon administration of roflumilast (mean ∆PD, −4.2 ± 1.0 mV) compared to vehicle (0.3 ± 0.7 mV, *P* < 0.001). Isc analysis of tracheal tissue excised from mice of the same genotype confirmed this effect in the lower airways (Fig. 1b), where mean ∆Isc was 53.8 ± 20.3 μA/cm^2^ with roflumilast vs. 10.4 ± 6.9 μA/cm^2^ with vehicle (*P* < 0.001). Roflumilast-stimulated Isc was sensitive to inhibition by CFTR_Inh_-172 (165.2 ± 31.4 μA/cm^2^). Together, these data indicate that roflumilast robustly activates CFTR in normal mice both in vivo and ex vivo. Note the concentrations used are clinically relevant [25] and were based on the maximum efficacy seen in prior in vitro studies using respiratory epithelial cell culture [20].

### Roflumilast acutely activates CFTR-mediated ion transport in cigarette smoke-exposed and air control mice

We have previously reported that normal (non-CF) A/J mice exposed to cigarette smoke via a nose-only exposure system twice daily for five weeks exhibit significantly diminished CFTR-mediated ion transport in the nose, trachea, and intestinal epithelia [9]. We next used this mouse model of cigarette smoke-induced CFTR dysfunction to better define the link between roflumilast and CFTR activation in the treatment of COPD. Following the exposure period, mice were treated with roflumilast (30 nM) infused acutely onto the nasal surface. As shown in the representative NPD tracing (Fig. 1c) and summary data (Fig. 1e), roflumilast perfusion in the setting of amiloride (100 μm), used to block sodium ion transport through epithelial sodium channels (ENaC), induced a rapid increase in chloride transport in air-exposed mice (−3.9 ± 0.8 mV) that significantly exceeded levels observed upon perfusion of vehicle (0.3 ± 0.7 mV, *P* < 0.01). Roflumilast also stimulated chloride transport in smoke-exposed mice (−2.0 ± 0.4 mV), although the smoke exposure diminished the stimulator effect of roflumilast in the nasal airways (*P* < 0.05). In contrast, in the lower airways, reduced stimulation by roflumilast was not observed in smoke exposed mice, as demonstrated by Isc analysis of excised tracheal segments (roflumilast air, 131.3 ± 29.3 μA/cm^2^ and roflumilast smoke, 147.7 ± 38.0 μA/cm^2^ Vs. vehicle air, 10.4 ± 7.0 μA/cm^2^, *P* < 0.01; Fig. 1d, f). Overall, these data indicate that roflumilast stimulates chloride transport in the nose and trachea of both smoke- and air-exposed mice in a CFTR-dependent manner.

### Chronic roflumilast oral administration reverses CFTR dysfunction in cigarette smoke-exposed mice

**Fig. 2:**
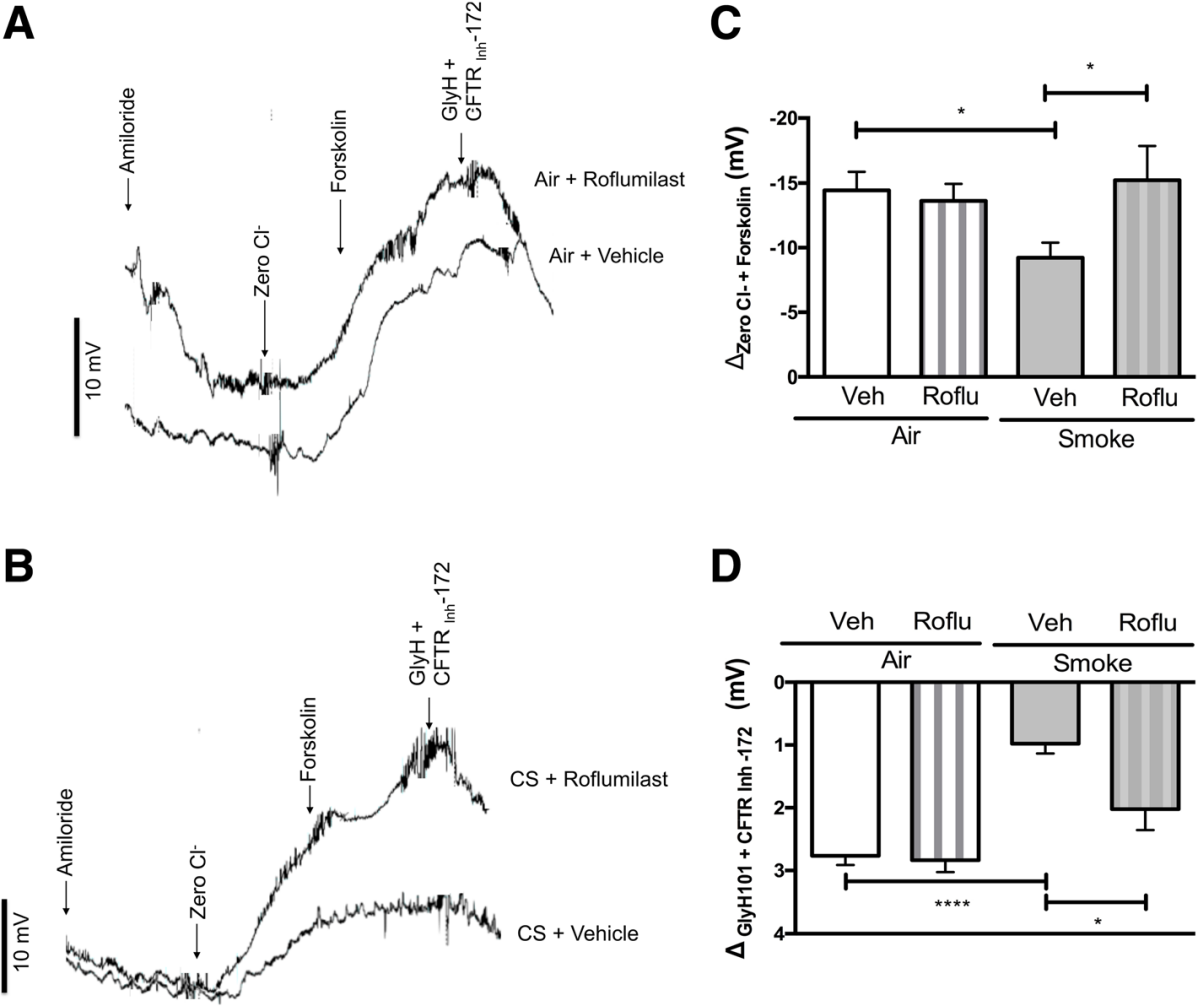
Effect of oral roflumilast co-administration in cigarette smoke-exposed mice. Non-CF A/J mice were treated with roflumilast (5 mg/kg/d) once daily via oral gavage over a five-week exposure period of cigarette smoke or air delivered through a nose-only exposure system. **a**, **b** Representative tracings of nasal potential difference (NPD) measurements of air control mice treated with vehicle or roflumilast (**a**) and cigarette smoke exposed mice treated with vehicle or roflumilast (**b**). **c** Mean forskolin-stimulated change in NPD upon sequential perfusion of Ringer’s solution, Ringer’s solution with amiloride (100 μM), chloride-free Ringer’s alone, and chloride-free Ringer’s with forskolin (20 μM). **d** Mean change in NPD upon perfusion of CFTR_Inh_-172 (10 μM). N = 5–13 mice/condition. *P < 0.05. ****P < 0.0001. Roflu = roflumilast, Veh = vehicle, CS = cigarette smoke exposed

Noting that once-daily oral administration of roflumilast is indicated for use in patients with COPD and chronic bronchitis [26], the target population of CFTR modulator therapy in COPD [19, 27, 28], we evaluated the effect of roflumilast administered to mice by oral gavage over time. Oral roflumilast (5 mg/kg/d) or vehicle was co-administered once daily with smoke or air exposure over five weeks. At the end of the treatment period, as anticipated, cigarette smoke alone reduced CFTR-dependent chloride transport to 64% of air exposure levels (smoke, −9.2 ± 1.2 mV vs. air, −14.5 ± 1.4 mV, *P* < 0.05; Fig. 2a-c). While oral roflumilast did not confer CFTR activation over vehicle-treated mice after air exposure (−13.6 ± 1.3 mV; Fig. 2a, c), likely because CFTR was maximally stimulated by perfusion of chloride-free forskolin by the NPD technique itself, CFTR activity in roflumilast-treated smoke-exposed mice was −15.2 ± 2.7 mV, marking a complete and significant (P < 0.05) reversal of the deleterious effects of smoke on CFTR ion transport (−9.223 ± 1.16 mV), which was substantially diminished (Fig. 2b, c) and consistent with prior literature [10, 17, 18]. Potential difference in both smoke and air-exposed mice treated with roflumilast was diminished after addition of CFTR_Inh_-172 (smoke, 2.02 ± 0.3 mV and air, 2.83 ± 0.2 mV), underscoring specificity of effect to CFTR (Fig. 2d). In vehicle-treated mice, CFTR_Inh_-172 elicited significantly lower reductions in potential difference in the presence of smoke compared to air exposure (smoke, 0.97 ± 0.1 mV vs. air, 2.76 ± 0.1 mV, *P* < 0.0001), indicative of lower baseline CFTR activity. Overall, these data indicate that chronic oral roflumilast treatment activates CFTR and reverses acquired CFTR dysfunction in vivo, and may explain its ability to improve health in patients with chronic bronchitis.

### Roflumilast does not exacerbate secretory diarrhea caused by cigarette smoke

**Fig. 3:**
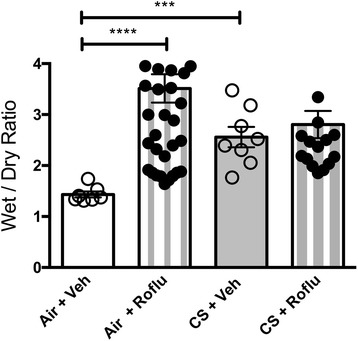
Effect of oral roflumilast co-administration in cigarette smoke-exposed mice on intestinal fluid secretion. Relative wet/dry ratio of stool sample collected from non-CF A/J mice treated with roflumilast (5 mg/kg/d) or vehicle once daily via oral gavage over a five-week exposure period of cigarette smoke or air delivered through a nose-only exposure system. *N* = 7–50 samples/condition, ****P < 0.0001

Our previous studies demonstrated that roflumilast increases fluid secretion in isolated murine intestinal segments in a CFTR-dependent manner, suggesting a mechanism underpinning non-infectious diarrhea seen with roflumilast therapy [20]. Roflumilast-induced diarrhea was apparent during the second week of oral administration in mice as evidenced by moderate perianal staining of the coat in drug-treated, but not vehicle-administered, mice. To quantify the effect of roflumilast on secretory diarrhea in vivo in the context of cigarette smoke, we measured the wet/dry ratio of stool specimen collected from mice following five weeks of oral co-administration of roflumilast (5 mg/kg/d) or vehicle with cigarette smoke or air exposure (Fig. 3). In air-exposed mice, roflumilast more than doubled the water content of stool specimen (roflumilast, 3.1 ± 0.2 vs. vehicle, 1.4 ± 0.1, *P <* 0.0001), corroborating our ex vivo findings [20]. Interestingly, exposure to cigarette smoke increased the wet/dry ratio of specimen to a level (2.6 ± 0.2) beyond which roflumilast had minimal additional effect (2.8 ± 0.3); given that the use of oral CFTR inhibitors that are not systemically absorbed has been proposed as an approach for offsetting roflumilast-associated diarrhea [29, 30], this finding presents the possibility that this strategy may be an effective approach worth evaluating prospectively, even in the background of cigarette smoking.

## Discussion

Multiple lines of evidence from our laboratory and others suggest that CFTR dysfunction can be acquired due to cigarette smoking and promotes COPD pathogenesis [9–12, 16–19]. Our prior studies of cigarette smoke exposure in non-CF A/J mice have provided key evidence underscoring the causal relationship between smoking and CFTR abnormalities, demonstrating significant decrement in CFTR activity in the nasal airway (25–35% of air control), tracheal tissue (50–60%), and intestinal tissue (80–90%) after five weeks of smoke exposure [9]. We observed a similar effect in these studies, in which the same smoke exposure in mice reduced CFTR activity in the nose to 64% of that seen in air controls. The magnitude of these decrements is comparable to those observed in the upper airway [10, 16], lower airway [12], and intestine [9] of cigarette smokers, as well upon treatment of airway cells with cigarette smoke [10, 16–18]. As such, this mouse model of cigarette smoke exposure is highly informative for evaluating the effects of roflumilast on CFTR-mediated ion transport in vivo, paving the way for further investigation with more complex animal models that exhibit bronchitis upon smoke exposure [31].

We have previously shown that roflumilast activates CFTR-mediated chloride transport in smoke-exposed primary HBE cells, as well as improves airway surface liquid depth compromised by smoke exposure [20]. These effects are conferred through a cAMP-dependent pathway [20], consistent with the known function of PDE4 inhibitors in augmenting intracellular cAMP levels and stimulating CFTR activity in airway epithelia [32–37]. Based on these findings, we hypothesized that roflumilast could improve CFTR function in the mouse model of cigarette smoke exposure. As assessed through NPD measurements, acute roflumilast activated CFTR in mice exposed to cigarette smoke for five weeks and, when co-administered by oral gavage over the exposure period, completely abrogated the detrimental effect of smoke on CFTR function. Potential difference readings in the nasal airway are reflective of levels in the lower airway [24], in line with our data demonstrating that roflumilast increased CFTR-mediated ion transport in tracheal tissue excised from smoke-exposed animals.

Interestingly, roflumilast acutely augmented CFTR-dependent ion transport in normal air control mice. This finding complements data reported in a recent publication demonstrating that the CFTR potentiator ivacaftor increased wild-type CFTR activity even in COPD patients who did not have diminished baseline CFTR activity at study inception [19], although the small sample size and short duration of the study did not allow for detection of association with changes in lung function. Although ivacaftor and roflumilast stimulate CFTR through different mechanisms [20, 38–40], it remains plausible that supranormal stimulation of CFTR activity by these and other agents in the context of fully functional CFTR may confer clinical benefit. A pilot study of ivacaftor benefited CFTR activity in some of the patients tested, and was associated with improved bronchitis symptom scores [19]. Additional studies that capture baseline CFTR function and evaluate the effect of ivacaftor- and roflumilast-mediated CFTR activation on clinical outcomes will be highly informative for enhanced understanding to this regard.

Non-infectious diarrhea is observed in nearly 10% of COPD patients who receive roflumilast therapy [26] and may be due, at least in part, to the ability of roflumilast to elicit CFTR-mediated fluid secretion in intestinal tissue [20]. Reminiscent of clinical observations, our studies demonstrate an elevated wet/dry ratio in stool samples collected from mice after oral roflumilast treatment over five weeks. Intriguingly, cigarette smoke alone caused secretory diarrhea to a level that was not further exacerbated by roflumilast did not notably exacerbate. Noting the evidence associating cigarette smoke with impaired CFTR function, even in the intestine [9], it is likely that this effect was mediated through a CFTR-independent mechanism. Nevertheless approaches currently being developed to treat diarrhea via CFTR inhibition by oral, non-bioavailable molecules [29, 30] may be of potential benefit in a subpopulation of patents who are cigarette smokers and experience diarrhea, particularly those who are intolerant of roflumilast, although further studies are needed to confirm these relationships.

While smoke-exposed mice are of great utility for evaluating the effect of roflumilast on CFTR function, this and other mouse models of COPD do not demonstrate mucus retention and other characteristics of chronic bronchitis, therefore precluding corresponding studies that can elucidate the relationship between roflumilast, CFTR, and therapeutic benefit [41], a key limitation. A ferret model of cigarette smoke-induced COPD that exhibits chronic mucus hypersecretion, goblet cell hyperplasia, and other features of chronic bronchitis was recently developed and will be instrumental in overcoming this important shortcoming in future studies [31].

## Conclusion

In conclusion, roflumilast reverses smoke-induced CFTR dysfunction and increases intestinal fluid in mice in a smoke-dependent manner, providing the first in vivo evidence that roflumilast may be functioning as a CFTR activator, which could explain its beneficial effects in COPD patients with bronchitis. By showing that roflumilast effectively rescues chloride transport in this model, our findings can be readily adopted to support clinical data regarding the effects of roflumilast in human subjects.

## Abbreviations

cAMP: cyclic adenosine monophosphate
CF: Cystic fibrosis
CFTR: Cystic fibrosis transmembrane conductance regulator
COPD: Chronic obstructive pulmonary disease
ENaC: Epithelial sodium channel
Isc: Short-circuit current
NPD: Nasal potential difference
PDE4: Type 4 cyclic nucleotide phosphodiesterase
PKA: Protein kinase A
